# STAT3 Target Genes Relevant to Human Cancers

**DOI:** 10.3390/cancers6020897

**Published:** 2014-04-16

**Authors:** Richard L. Carpenter, Hui-Wen Lo

**Affiliations:** 1 Division of Surgical Sciences, Department of Surgery, Duke University School of Medicine, Durham, NC 27710, USA; E-Mail: richard.carpenter@duke.edu (R.L.C.); 2 Duke Cancer Institute, Duke University School of Medicine, Durham, NC 27710, USA

**Keywords:** STAT3, cancer, gene transcription

## Abstract

Since its discovery, the STAT3 transcription factor has been extensively studied for its function as a transcriptional regulator and its role as a mediator of development, normal physiology, and pathology of many diseases, including cancers. These efforts have uncovered an array of genes that can be positively and negatively regulated by STAT3, alone and in cooperation with other transcription factors. Through regulating gene expression, STAT3 has been demonstrated to play a pivotal role in many cellular processes including oncogenesis, tumor growth and progression, and stemness. Interestingly, recent studies suggest that STAT3 may behave as a tumor suppressor by activating expression of genes known to inhibit tumorigenesis. Additional evidence suggested that STAT3 may elicit opposing effects depending on cellular context and tumor types. These mixed results signify the need for a deeper understanding of STAT3, including its upstream regulators, parallel transcription co-regulators, and downstream target genes. To help facilitate fulfilling this unmet need, this review will be primarily focused on STAT3 downstream target genes that have been validated to associate with tumorigenesis and/or malignant biology of human cancers.

## 1. Introduction

Signal transducer and activator of transcription-3 (STAT3), also known as acute phase response factor (APRF), is a DNA-binding transcription factor [1,2]. STAT3 belongs to the STAT family of transcription factors consisting of seven proteins, namely, STAT1, STAT2, STAT3, STAT4, STAT5a, STAT5b and STAT6. Activated STAT3 translocates into the cell nucleus and binds to the interferon-gamma activated sequence (GAS) within target gene promoters to regulate gene transcription [3]. When cells are exposed to cytokines and growth factors, cytoplasmic STAT3 becomes phosphorylated at tyrosine-705 (Y705) [4]. Classically, phosphorylated STAT3 can homodimerize, as well as heterodimerize with STAT1, and then undergo nuclear transport. However, it is now recognized that unphosphorylated STAT3 also undergoes nucleo-cytoplasmic shuttling and up regulates genes, a large portion of which overlap with those up regulated by phosphorylated STAT3 [5,6]. Phosphorylation of the STAT3 Y705 residue can be induced by cytokines (IL-6, IFN-α), growth factors (EGFR, EGFRvIII, HER2 and PDGFR) and non-receptor tyrosine kinases (Src and all the JAK family proteins) [7,8,9]. Inactive JAK proteins constitutively bind G-protein coupled receptors, IL-Rs, LIF-R, gp130, and undergo autophosphorylation and activation upon receptor activation. JAK2 activation can be mediated by EGFR and EGFRvIII. STAT3 can also be phosphorylated at Ser727 by ERK, CDK1, ATM and ATR [10]. Evidence to date suggests that Ser727 phosphorylation may be essential for the maximal transcriptional activity of Y705-phosphorylated STAT3 although the true impact of S727 phosphorylation remains somewhat controversial. For example, a study [11] showed that STAT3 activation by a phosphomimetic S727 induced prostate cancer formation independent of Y705 phosphorylation. Dephosphorylation of Y705-phosphorylated STAT3 can occur through protein tyrosine phosphatases, SHP-1/2, Meg2 and protein tyrosine phosphatase delta (PTPRD). The STAT3 pathway can be inhibited by suppressor of cytokine signaling 3 (SOCS3) and protein inhibitors of activated STATs (PIAS) [12,13,14].

STAT3 is frequently constitutively activated in many human cancers [15,16,17,18,19]. In cancerous cells, STAT3 constitutive activation is common, which is likely due to the aberrant activity of STAT3’s upstream signaling pathways, such as, EGFR, HER2, Src and JAK2. Tumorigenic STAT3 activation has been frequently linked to more malignant cancer behaviors, including growth, epithelial-mesenchymal transition, migration, invasion and metastasis. STAT3 activation is also associated with tumor survival and therapeutic resistance. In immune cells, STAT3 activation often leads to immune suppression and evasion. Together, STAT3 activation in both tumors and immune cells contributes to several malignant phenotypes of human cancers and to compromised anti-cancer immunity, together leading to poor clinical outcomes.

Despite the findings of STAT3 hyperactivation in many cancers, the role of STAT3 in oncogenesis is still unclear and is likely dependent on tumor type and cellular context [20]. Given its ability to regulate both oncogenes and tumor suppressor genes, STAT3 has been reported to promote and inhibit oncogenesis. STAT3 has been shown to induce cancers of the breast [21,22], prostate [11] and skin [23,24]. STAT3 has been reported to transform bone marrow cells into T cell leukemia in a mouse model [20]. In contrast to its positive impact on oncogenesis, activated STAT3 has been shown to suppress c-Myc-mediated transformation of mouse embryonic fibroblasts [20]. STAT3 can promote EGFRvIII-induced glial transformation by forming a nuclear complex with EGFRvIII [25]. The oncogenic role of STAT3 in gliomas is consistent with the observation that STAT3 activation is rarely detected in normal brain tissues [15,17]. In contrast, in PTEN-proficient mouse astrocytes, STAT3 appeared to behave as a tumor suppressor while simultaneous suppression of PTEN and STAT3 led to astrocyte malignant transformation [25]. 

The broad influence STAT3 has on cellular function can be attributed to the numerous gene targets that have been identified for STAT3 (Table 1). STAT3 can indirectly regulate genes by mediating expression of other transcription factors or physical association with other transcription factors to enhance or suppress their function in gene regulation. This review will discuss direct STAT3 target genes, which are only considered direct if there is evidence of STAT3 binding either by chromatin immunoprecipitation (ChIP) or electrophoretic mobility shift assay (EMSA). Detection of STAT3 binding by ChIP shows STAT3 binding in cells whereas evidence by EMSA is *in vitro* but results from either method will be considered as evidence for direct binding. We will discuss the regulation of STAT3 target genes that result in the tumor supporting functions of STAT3, including several transcription factors, apoptosis, tumor immune surveillance, metastasis, tumor angiogenesis, and oncogenic cell signaling. In addition, the direct STAT3 target genes where the outcome is the tumor suppressing functions of STAT3 will be detailed. Finally, STAT3 regulation of genes that may play dual roles in tumor growth will be examined. 

**Table 1:** Gene regulation by STAT3.

STAT3-Regulated Genes		Direct Binding	STAT3 Binding Site(s)	Cell/Tissue Type(s)	Species	Reference
Tumor Supporting Functions of STAT3						
*Transcription Factors*						
↑	c-Fos	EMSA/ChIP	−348 to −339 bp	HepG2, A431 Cells	Human	[19,26,27]
↑	HIF-1α	EMSA/ChIP	−363 to −355 bp	A2058, v-Src-3T3 Cells, B16 Tumors	Human Murine	[28]
↑	c-Myc	EMSA/ChIP	+84 to +115 bp	HepG2, BAF-G277, KT-3, CCE ES Cells	Human Murine	[29]
↑	Sox2	ChIP	−5.7 to −3.3 kb −528 to +238 bp	CCE ES Cells	Murine	[30]
↑	Nanog	ChIP	−871 to −585 bp	Mouse Embryonic Cells	Murine	[31]
↑	Twist	ChIP	−116 to −107 bp −103 to −96 bp	A431 Cells	Human	[19]
↑	Zeb1	ChIP	−310 to −130 bp	SW1116, LoVo Cells	Human	[32]
↓	p53	EMSA/ChIP	−128 bp	NIH-3T3, MEF Cells	Murine	[33]
↑	Oct-1	ChIP	−3.5 to −2.5 kb	Eca-109 Cells	Human	[34]
*Apoptosis and Proliferation*						
↑	Bcl-2	ChIP	−1022 to −1002 bp	Hela Cells	Human	[35]
↑	Mcl-1	EMSA	−94 to −86 bp	U266, v-Src-3T3 Cells	Human Murine	[36,37]
↑	Bcl-xL	ND	−600 to 0 bp	U266 Myeloma, NIH-3T3 Cells	Human Murine	[38]
↑	Survivin	EMSA/ChIP	−1174 to −1166 bp −1095 to −1087 bp	MDA-MB-453, NIH-3T3 Cells	Human Murine	[39]
↓	Fas	ChIP	−460 to −240 bp	Myeloma Cells	Human	[40]
↑	Hsp70	EMSA/ChIP	−122 to −90 bp	VSM, HeLa Cells	Human	[41]
↑	Hsp90α	ChIP	−1642 to −1485 bp	Jurkat Cells	Human	[42]
↑	Hsp90β	EMSA	−643 to −623 bp	VSM Cells	Human	[41]
↑	Cyclin-D1	EMSA/ChIP	−984 bp, −568 bp, −475 bp, −239 bp	293T, 3YI, NIH-3T3, 2fTG Cells	Human Murine	[43,44,45]
*Immune Suppression and Inflammation*						
↑	IL-10	EMSA/ChIP	−120 to −111 bp	RPMI-8226 B Cells	Human	[46]
↑	IL-23	ChIP	−1159 to +160 bp	B16 Tumors	Murine	[47]
↑	TGF-β	ChIP	−3155 to −2515 bp	CD4+ T Cells	Murine	[48]
↑	COX-2	ChIP	−134 to −127 bp	U87MG Cells	Human	[49]
*Metastasis*						
↑	MMP-1	EMSA/ChIP	−79 to −42 bp	T24, HT-29 Cells	Human	[50]
↑	MMP-2	EMSA	−1657 to −1620 bp −625 to −601 bp	C4 K1735 Cells	Murine	[51]
↑	MMP-3	ChIP	−410 to −110 bp	HBVE Cells	Human	[52]
↑	MMP-9	ChIP	−942 to −934 bp	MCF7 Cells	Human	[53]
↑	Fascin	ChIP	−1095 to −1067 bp −975 to −948 bp	4T1, MDA-MB-231 Cells	Human Murine	[54]
↑	Vimentin	EMSA/ChIP	−757 to −749 bp	MDA-MB-231, C2C12 Cells	Human Murine	[55]
↑	RhoU	EMSA/ChIP	−1067 to −324 bp	MEF Cells	Murine	[56]
↑	ICAM-1	EMSA/ChIP	−76 to −66 bp −175 to −97 bp	HepG2, BV2 Cells	Human Murine	[57]
↑	NGAL	ChIP	−170 bp	Primary Macrophages	Human	[58]
↑	POMC	EMSA	−399 to −374 bp	AtT20 Cells	Murine	[59]
↑	SAA1	EMSA/ChIP	−226 to +24 bp	HepG2 Cells	Human	[60]
*Angiogenesis*						
↑	VEGF-A	EMSA/ChIP	−848 bp	v-Src-3T3 Cells	Murine	[61]
↑	bFGF	ChIP	−997 to −989 bp	HUVEC	Human	[62]
↑	HGF	EMSA/ChIP	−149 bp, −110 bp	SP1, RINm5F Cells	Murine	[63,64]
*Cell Signaling*						
↑	AKT	ChIP	Proximal −2.2 kb	293 Cells	Human	[65]
↑	PIM-1	ChIP	−934 to −905 bp	Microglial Cells	Murine	[66]
↑	TNF-R2	ChIP	−1578 bp, −364 bp	SW480 Cells	Human	[67]
↑	S1P-R1	EMSA/ChIP	−588 bp	MB49 Cells, B16 Tumors	Murine	[68]
↑	MUC-1	EMSA/ChIP	−503 to −495 bp	T74D, ZR-75-1 Cells	Human	[69,70]
*Transcription Factors*						
↑	FOXO1	ChIP	−515 bp	CD4+ T Cells	Murine	[71]
↑	FOXO3A	ChIP	+196 bp	CD4+ T Cells	Murine	[71]
↑	Foxp3	ND	Intron 1	293 Cells	Human	[72]
↓	Necdin	EMSA/ChIP	−588 bp	v-Src-3T3	Murine	[73]
*Survival and Metastasis*						
↑	p21^CIP1/WAF1^	EMSA/ChIP	−4183 bp −2540 bp −640 bp	MG63, A431, HT-29, WiDr, HepG2 Cells	Human	[74,75,76]
↑	PI3K p50α	ChIP	−276 bp	Mammary Gland	Murine	[77]
↑	PI3K p55α	ChIP	−624 bp	Mammary Gland	Murine	[77]
*Tumor Immune Surveillance*						
↑↓	IL-6	ChIP	−73 to −54 bp	NIH-3T3, CT26	Murine	[78,79,80]
↑↓	TNF-α	ND	−1452 bp	Macrophages, SCK1	Murine	[80,81]
↑↓	IFN-γ	ChIP	105 to 542 bp	T Cells	Human Murine	[78,80,82]
↑↓	RANTES	EMSA/ChIP	−120 to −1 bp	PC3, NIH-3T3 Cells	Human Murine	[78,80,83]
↑	CRP	EMSA	−112 to −105 bp	Hep3B Cells	Human	[84]
↑	STAT1	ChIP	−604 to −596 bp −444 to −435 bp −363 to −356 bp −246 to −239 bp	MDA-MB-468 Cells	Human	[85]
↑	RORγt	ChIP	1st Intron	TH17 Cells	Murine	[86]
↑	RORα	ChIP	1st Intron	TH17 Cells	Murine	[86]
↑	BATF	ChIP	2nd Intron	TH17 Cells	Murine	[86]
↑	IRF4	ChIP	Proximal Promoter	TH17 Cells	Murine	[86]
↑	IL-6Rα	ChIP	1st Intron	TH17 Cells	Murine	[86]
↑	IL-23R	ChIP	UD	TH17 Cells	Murine	[86]
↑	IL-17A	ChIP	−144 bp	TH17 Cells	Murine	[86,87]
↑	IL-17F	ChIP	−309 bp −326 bp	TH17 Cells	Murine	[86,87]
*Other*						
↑	TIMP-1	EMSA/ChIP	−49 to −41 bp	HepG2, WI38, CD4+ T Cells	Human Murine	[88,89]
↑	JunB	EMSA	−196 to −91 bp	HepG2 Cells	Human	[26,90]
↑	iNOS	EMSA/ChIP	−142 to −130 bp −84 to −60 bp	A431 Cells	Human	[18]
↑↓	CDC25A	ChIP	−222 to +58 bp	HepG2, Saos Cells	Human	[91]

* Direct binding required evidence by ChIP or EMSA; ND = Not determined; UD = Undescribed.

## 2. Tumor Supporting Functions of STAT3

STAT3 can promote tumor growth by mechanisms within the tumor cell as well as in the tumor microenvironment. As will become clear, STAT3 can directly regulate genes leading to multiple tumor promoting processes from cell survival, invasion, angiogenesis, and immune escape. In addition, STAT3 can regulate other transcription factors that also support tumor growth, thus STAT3 can also indirectly support tumor growth by promoting survival, angiogenesis, metastasis, and even pluripotency. 

### 2.1. Transcription Factors

There is a broad range of transcription factors that are regulated by STAT3 and none likely have as many broad functions as c-Fos and HIF-1α. Cellular Fos (c-Fos) is a member of the activating protein-1 (AP-1) transcription factor complex composed of dimers from the Jun (c-Jun, JunB, and JunD) and Fos (c-Fos, FosB, Fra1, and Fra2) families. AP-1 is frequently up regulated in several cancer types and also has broad influences supporting tumor inflammation, angiogenesis, and suppression of apoptosis among others [92]. STAT3 has been shown to up regulate c-Fos expression in both hepatocellular and epidermoid carcinoma cells and results indicated this was a result of direct interaction of STAT3 with the c-Fos gene promoter [19,26,27]. Hypoxia inducible factor 1 alpha (HIF-1α) combines with HIF-1β, which is constitutively expressed, to form a dimer that can regulate a multitude of genes that result in tumor growth and progression. The HIF-1 complex is up regulated in multiple cancer types and promotes tumor growth by inducing genes that promote angiogenesis, invasion, and alters cell metabolism to be more characteristic of cancer cells [93]. HIF-1α can be regulated transcriptionally but also by post-translational mechanisms. STAT3 has been shown to increase HIF-1α levels by extension of protein half-life but also induction of HIF-1α transcription in human and mouse melanoma cells [28,94]. The *HIF1A* gene was found to contain STAT3 binding sites and STAT3 was confirmed to directly bind the *HIF1A* gene promoter [28]. While STAT3 can directly regulate several genes that support tumor growth, these effects are compounded by the upregulation of c-Fos and HIF-1α expression. STAT3, c-Fos, and HIF-1 alone can support a multitude of processes that promote initiation and progression of tumors.

STAT3 has been shown to be dysregulated in cancer stem cells [95]. Thus, it is not surprising that STAT3 targets several transcription factors that can promote or support stemness. The genes for c-Myc, Sox2, and NANOG have been shown to be intimately involved in the pluripotency of cells [96]. Expression of STAT3 has been shown to up regulate all three of these transcriptions factors [29,30,31]. ChIP assays have confirmed that STAT3 can directly associate with the promoter of all three of these genes in embryonic stem cells [29,30,31]. In addition, HIF-1 has also been shown to support cancer stem cell renewal [97]. Thus, STAT3 can directly up regulate multiple transcription factors that can reprogram cells to induce and sustain pluripotency supporting a cancer stem cell phenotype.

A key step in the metastasis of carcinomas is the conversion of epithelial cancer cells to mesenchymal cancer cells called epithelial-to-mesenchymal transition (EMT). A vital point in EMT is the down regulation of E-cadherin expression, which ultimately promotes intercellular dissociation allowing cell migration, resistance to anoikis, and resistance to chemotherapy among others [98]. This advancement of the EMT program is achieved by expression of transcriptional repressors. Our lab has shown that STAT3 mediates EGF-induced expression of the repressor Twist in epidermoid and mammary carcinoma cells [19]. We found STAT3 binding sites within the *TWIST1* promoter and showed direct binding of STAT3 via ChIP [19]. In addition to TWIST, STAT3 has also been shown to up regulate expression of ZEB1, another E-cadherin repressor [32]. They observed STAT3 could also directly associate with the *ZEB1* promoter in colorectal carcinoma cells [32]. These studies implicate STAT3 as an inducer of EMT and ultimately tumor progression toward metastasis.

STAT3 also has an indirect effect on cell proliferation by regulating Oct-1 and p53. Octamer transcription factor-1 (Oct-1) is a widely-expressed transcription factor that can induce expression of genes promoting anaerobic metabolism but also promotes cell proliferation [99,100]. Knockdown of STAT3 suppressed Oct-1 expression in esophageal squamous cancer cells [34]. STAT3 was also shown to directly bind the gene promoter for Oct-1 to regulate its expression [101]. Upregulation or activation of p53 occurs in response to many cell stressors from genetic abnormalities to oxidative stress and can potently induce apoptosis and cell cycle arrest [102]. Induction of STAT3 by expression of v-Src has been shown to suppress p53 levels and this was reversed with expression of a STAT3 dominant negative [33]. The gene promoter for p53 contained multiple STAT3 predicted binding sites but STAT3 could only directly associate with one of these sites (−128 bp) to suppress p53 gene expression [33]. The expression of Oct-1 coupled with down regulation of p53 levels, mediated by direct STAT3 regulation, results in a higher potential for progression through the cell cycle and uncontrolled proliferation characteristic of cancer cells.

### 2.2. Apoptosis and Proliferation

Apoptosis is regulated by two primary pathways: the extrinsic and intrinsic pathways. The extrinsic pathway is initiated by ligands binding to death receptors (e.g., Fas ligand and Fas), which directly activate caspases to initiate the apoptosis program. The intrinsic pathway is regulated at the mitochondria whereby the balance of pro-apoptotic (e.g., Bim, Bad, Bik, PUMA, and NOXA) and anti-apoptotic (e.g., Bcl-2, Bcl-xL, Mcl-1, A1, and Bcl-w) proteins from the Bcl-2 protein family determine the integrity of the mitochondrial membrane. Upon accumulation of pro-apoptotic proteins, mitochondrial outer membrane permeabilization (MOMP) occurs resulting in release of cytochrome *c* from the mitochondria forming the apoptosome to activate caspases and initiate apoptosis. Several of these proteins have been discovered to be STAT3 target genes.

Anti-apoptotic Bcl-2 proteins, such as Bcl-2 and Mcl-1, interact with the pore-forming proteins Bax and Bak preventing their induction of MOMP and ultimately apoptosis. After induction of both Bcl-2 and STAT3 with phospholipase D in HeLa cells, the Bcl-2 gene was found to contain STAT3 binding sites [35]. STAT3 was found to directly associate with the Bcl-2 gene promoter and up regulate Bcl-2 expression thereby suppressing apoptosis in these cells [35]. Binding sites for STAT3 were also found in the gene promoter for Mcl-1 and inhibition of STAT3 resulted in decreased Mcl-1 levels in peripheral leukocytes [8,103,104]. EMSA experiments later showed that STAT3 can directly bind these STAT3 elements within the *MCL1* promoter and drive gene activation in LGL leukemia cells [37]. Another anti-apoptotic protein, Bcl-xL, was also seen to be up regulated with STAT3 expression [38]. Serial truncation of the Bcl-xL gene promoter revealed the proximal region of the promoter was required for STAT3-induced activation of the Bcl-xL gene but direct binding was not assessed [38].

Caspases are the terminal effectors of the apoptosis program and can be directly activated as well as directly inhibited. Aside from the indirect regulation of caspases by the Bcl-2 protein family, caspases can be directly activated by death receptors. One such receptor is the Fas receptor, which binds the Fas ligand and uses the intracellular death domain to directly activate caspases to initiate apoptosis. Expression of a dominant-negative STAT3 mutant induced expression of Fas and, in addition, cells lacking c-Jun were responsive to Fas ligand indicating presence of the receptor [40]. The Fas gene promoter was found to contain binding sites for both STAT3 and AP-1 and mutation of both of these sites increased Fas gene activation greater than mutation of either site alone indicating these two sites likely work together to repress Fas expression [40]. Both EMSA and ChIP assays confirmed that STAT3 and c-Jun bind the Fas gene (*TNFRSF6*) in a complex in order to repress Fas expression [40]. Caspases can also be directly inhibited by inhibitor of caspases (IAPs), such as survivin, which interact with and suppress activation of caspases to block apoptosis. STAT3 expression has been shown to induce expression of survivin and suppress apoptosis [39]. Both EMSA and ChIP assays confirmed that STAT3 could directly associate with the survivin promoter to induce expression [39].

Heat shock proteins (Hsps) are typically expressed in response to acute cell stress to ensure proteins are folded correctly and to maintain integrity of existing proteins. Hsp proteins are commonly overexpressed in multiple malignant cell types and support tumor growth by protecting oncogenic proteins from degradation [105]. It was observed that thrombin could induce expression of Hsp70 and Hsp90β in vascular smooth muscle cells (VSMCs), which was inhibited by suppression of STAT3 signaling [41]. Experiments using EMSA identified that both STAT3 and STAT1 could bind to a sequence in the Hsp70 gene promoter as well as to a sequence in the Hsp90β gene promoter [41]. In addition, the Hsp90α gene promoter was found to contain the GAS binding sequence [42]. Investigators found that in quiescent Jurkat cells, STAT1 was constitutively bound to this GAS sequence in the Hsp90α gene promoter but heat shock induced binding of STAT3 and HSF1, in addition to STAT1, leading to increased Hsp90α mRNA levels [42]. IFN-γ treatment prevented additional binding of STAT3 and HSF1 to the Hsp90α GAS sequence inhibiting induction of Hsp90α expression with heat shock [42].

In addition to suppressing apoptosis, STAT3 also functions to up regulate proliferation by promoting entry to the cell cycle. The cell cycle is regulated by waves of expression of proteins that drive the cell cycle forward during each phase. In particular, the cyclin D proteins regulate the G_1_/S-phase transition as the cyclin D proteins (D1, D2, and D3) increase expression leading to interaction with and activation of cyclin dependent kinases 4 and 6 (CDK4/6), which phosphorylates the retinoblastoma (Rb) protein to initiate S-phase [106]. Thus, induction of cyclin D proteins, including cyclin D1, promotes entry into the S-phase and cell proliferation. Early studies showed that an active form of STAT3 could induce cyclin D1 whereas a STAT3 dominant negative could suppress Src-induced cyclin D1 expression [45]. A later study identified STAT3 binding sites within the cyclin D1 gene and observed direct association of STAT3 with the promoter and induction of proliferation [44]. Tumor growth is the result of uncontrolled cell proliferation due to flux through the cell cycle combined with suppression of normal cellular death signals. Thus far, STAT3 has been shown to directly affect both of these mechanisms by upregulating anti-apoptosis proteins as well as cyclin D1 giving substantial reason to those targeting STAT3 for suppressing tumor growth.

### 2.3. Immune Suppression and Inflammation

STAT3 has been elegantly shown to promote tumor evasion of the immune system [80,107]. STAT3 achieves promoting tumor immune evasion by directly upregulating expression of multiple cytokines that suppress immune function. In addition, STAT3 also up regulates the pro-inflammatory intracellular enzyme COX-2. STAT3 has been shown to indirectly affect several genes related to antigen presentation that cause immune suppression [107,108] but this discussion will focus primarily on genes that STAT3 regulates directly.

Interleukin-10 (IL-10) is an immunosuppressive cytokine that suppresses the production of pro-inflammatory cytokines such as TNF-α and IL-1 and reducing cell surface expression of antigen-presenting proteins. IL-10 is induced by STAT3 in human RPMI 8226.1 B cells [46,109,110]. It was later confirmed that STAT3 could directly associate with the *IL10* promoter in these cells [46]. IL-23 is another immune suppressing cytokine that has been shown to reduce tumor infiltration by CD8^+^ T cells and promote tumor angiogenesis [111]. IL-23 levels were seen to be reduced with knockdown of STAT3 and further examination revealed a STAT3 binding site on the IL-23 gene promoter [47]. A ChIP assay confirmed direct association of STAT3 with this promoter [47]. TGF-β is a cytokine that can suppress Th1 and Th2 cells in addition to its promotion of EMT on epithelial tumor cells. Introduction of an active STAT3 increased expression of TGF-β1 [48]. A search of the TGF-β1 gene found STAT3 binding sites within the promoter and ChIP assay confirmed direct association of STAT3 to the TGF-β1 gene promoter [48]. Thus, STAT3 has a very direct role in suppressing tumor immune surveillance and function by regulation of these cytokines.

Cyclooxygenase-2 (COX-2) is an inducible prostaglandin synthase that is involved in several pathological processes related to inflammation and is up regulated in several types of cancers [112]. Our lab observed increased COX-2 levels with the presence of nuclear EGFR in U87MG human glioblastoma cells [49]. COX-2 expression was further enhanced in these cells with co-expression of an active form of STAT3 [49]. We found a STAT3 target sequence in the COX-2 gene promoter and the STAT3-EGFR complex was found to associate directly with this promoter sequence to induce activation of the gene for COX-2 [49]. These results indicate a significant function of STAT3 is to suppress immune function and promote tumor survival. However, these do not entirely encompass the effect of STAT3 on immune function. Other roles are discussed in the section below on the dual roles of STAT3 in tumor growth.

### 2.4. Metastasis

Tumor metastasis indicates the spread and implantation of tumor cells from the primary tumor site to distant tissues. Tumor metastasis is generally associated with disease progression, decreased survival, and decreased response to therapy. There are many steps and requirements for tumor cell metastasis including detachment of tumor cells from primary tumors, invasion of these cells out of the primary tumor, entry to a transportation system (*i.e.*, lymphatic system), and implantation into the new tissue site. STAT3 has been shown to up regulate several genes that support multiple functions required for metastasis.

A key step in metastasis is invasion of cells out of the primary tumor site into neighboring tissues. In order for invasion to occur, there is often release of proteases from the tumor cell that degrade extracellular matrix allowing passage of the migrating cell. Proteases also play a role in angiogenesis as pro-angiogenic growth factors are often released from matrix degraded by proteases and they also degrade basement membrane for endothelial cell migration. The matrix metalloproteinase (MMP) family of proteases are zinc-dependent and are capable of degrading many types of extracellular matrix proteins and several family members are confirmed STAT3 target genes. MMP-1 was observed to be up regulated in a STAT3-dependent manner in human colorectal adenocarcinoma and urinary bladder transitional cell carcinoma cells [50,113]. The *MMP1* gene was found to contain a STAT3 and an AP-1 binding site and mutation of either the STAT3 or AP-1 site ablated STAT3-mediated gene activation [50,113]. Further investigation revealed both STAT3 and c-Jun were directly bound to the *MMP1* promoter in a manner dependent on c-Jun binding [50]. In addition to MMP-1, expression of an active STAT3 was also found to induce transcriptional activation of the gene promoter for MMP-2 in mouse melanoma cells [51]. The MMP-2 gene promoter contained several potential STAT3 binding sites and EMSA showed that STAT3 could directly bind the promoter [51]. STAT3 has been observed to induce activation of MMP-3 in human brain vascular endothelial cells [52]. Analysis of the *MMP3* gene revealed several STAT3 binding sites and a subsequent ChIP assay confirmed direct binding to the gene promoter by STAT3 [52]. Lastly, STAT3 can regulate the gene for MMP-9. In MCF7 human breast cancer cells, stable expression of an active STAT3 increased MMP-9 mRNA levels as well as transcriptional activation of the MMP-9 gene [114]. This study found STAT3 binding to the *MMP9* gene via EMSA and a later study confirmed this result using a ChIP assay [53,114].

Once extracellular matrix is cleared to allow cell migration, the cell requires upregulation of proteins to control the cytoskeleton to allow for cell migration. Fascin is one such protein as it is an actin-bundling protein that promotes tumor cell migration, invasion, and metastasis [115]. The human and mouse fascin gene promoter was found to contain a motif similar to the canonical STAT3 binding site [54]. Activation of STAT3 in murine 4T1 cells or human MDA-MB-231 cells induced expression of fascin [54]. Further analysis with a ChIP assay confirmed that STAT3 could directly associate with the gene promoter for fascin in both breast cancer cell types [54]. Vimentin is a type III intermediate filament and supports the structural integrity of the cell while providing flexibility to the cell to allow cell movement. Vimentin is often used as a marker for the mesenchymal phenotype and expression in epithelial tumor cells often indicates EMT and capabilities for cell migration. The vimentin gene promoter was found to contain a STAT3 binding site within the antisilencer element (ASE) and deletion of this region significantly reduced STAT3-induced transcriptional activation of the vimentin gene in MDA-MB-231 and mouse C2C12 cells [55]. Further analysis showed that STAT3 could directly bind these target sequences from the *VIM* promoter [55]. Lastly, STAT3 has been shown to regulate the GTPase RhoU, which acts on the actin cytoskeleton to stimulate filopodia formation and loss of RhoU inhibits cell migration [116,117]. Gene expression profiling of STAT3-null cells led to the identification of RhoU as a possible STAT3 target gene [56]. Treatment of mouse embryonic fibroblasts (MEFs) with gp130 cytokines induced RhoU mRNA levels whereas STAT3-null cells showed significantly lower RhoU expression [56]. EMSA experiments suggested STAT3 could bind multiple sequences in the RhoU gene promoter and two of these sites (−1,067 bp and −324 bp) were also confirmed binding sites by ChIP analysis [56].

Another protein that supports metastasis is intercellular adhesion molecular-1 (ICAM-1), a membrane adhesion molecule up regulated in inflammatory conditions [118]. ICAM-1 has been shown to have higher levels in tumor tissues and antibody targeting of ICAM-1 induces potent macrophage-dependent antimyeloma activity [118,119]. ICAM-1 supports metastasis by making it easier for ICAM-1-expressing tumor cells to separate from each other and invade surrounding tissue [120]. The ICAM-1 gene was identified as having an IL-6 response element [57]. It was seen that STAT3 forms a complex with c-Fos and c-Jun to bind and up regulate the *ICAM1* gene in HepG2 human hepatocellular carcinoma cells [57].

STAT3 can also regulate genes for secreted proteins that play a role in metastasis. Neutrophil gelatinase associated lipocalin (NGAL), or lipocalin 2, is a secreted glycoprotein that has an immune function to protect against bacterial infection [121]. In addition, it also has several tumor promoting functions including stabilization of MMP-9 and, therefore, promotion of cell invasion [121]. IL-10 has been shown to induce NGAL expression and gene activation in human primary macrophages, which was suppressed with inhibition of JAK/STAT signaling [58]. Analysis of the NGAL gene promoter showed a STAT3 binding site and mutation of this site (-170 bp) reduced NGAL gene activation by IL-10 [58]. A ChIP assay then confirmed STAT3 could directly associate with the NGAL gene promoter [58]. Another secreted protein, proopiomelanocortin (POMC), is a precursor peptide that gives rise to multiple derivative peptides such as α-MSH, a known promoter of the growth and metastasis of melanoma [122]. Leukocyte inhibitory factor (LIF) was observed to induce activation of the gene for POMC and murine pituitary tumor cells [59]. Knowing that LIF activates STAT3 signaling, further search of the gene for POMC showed a STAT3 binding site [59]. Deletion of this region reduced gene activation by LIF and EMSA experiments showed that STAT3 could directly bind this sequence [59].

Lastly, STAT3 has also been found to regulate the gene for serum amyloid A (SAA), which can interact with and alter the composition of the extracellular matrix to promote tumor initiation and progression [123]. The SAA proteins are also a family of acute-phase proteins that are at high levels in serum of cancer patients and may be a cancer biomarker [123]. Expression of STAT3 induced transcriptional activation of the SAA1 and SAA2 genes in HepG2 hepatocellular carcinoma cells whereas a STAT3 dominant negative suppressed their activation [60]. EMSA showed that STAT3 could bind sequences within the *SAA1* promoter and did so in a complex with the p65 subunit of NF-κB and the acetyltransferase p300 [60]. Subsequent analysis by ChIP assay confirmed that STAT3 was bound to the *SAA1* promoter in a complex with p300 [60].

Taken together, it appears that STAT3 up regulates many genes that support tumor metastasis through a variety of mechanisms. This facet of STAT3 biology likely contributes to the association of STAT3 with metastasis and a poor prognosis in multiple cancer types [16,124,125,126].

### 2.5. Angiogenesis

Tumors have high proliferation rates and metabolism. These large amounts of activity require a high demand for oxygen and nutrients. Once tumors reach approximately 2 mm in diameter, the center of the tumor becomes hypoxic, which leads to activation of tumor angiogenesis. Tumor angiogenesis is thought to be regulated by the balance of pro-angiogenic (e.g., VEGF-A and basic fibroblast growth factor) and anti-angiogenic (e.g., angiostatin and endostatin) factors present in the tumor microenvironment [127]. STAT3 has been shown to up regulate tumor angiogenesis by directly targeting multiple pro-angiogenic factors.

Vascular endothelial growth factor A (VEGF-A) is the most studied and most potent pro-angiogenic growth factor known. STAT3 expression induces VEGF-A protein levels in NIH-3T3 cells and promotes angiogenesis [61]. The gene for VEGF-A was found to have STAT3 binding sites and this study provided evidence that STAT3 can directly bind one of these sites to promote VEGF-A expression [61]. Basic fibroblast growth factor (bFGF) is another well-studied pro-angiogenic growth factor that activates fibroblast growth factor receptors (FGFRs) to promote endothelial cell proliferation and angiogenesis. STAT3 can up regulate bFGF expression in HUVEC cells in response to VEGF stimulation and evidence shows STAT3 can associate directly with the bFGF gene promoter [62]. STAT3 has also been shown to be activated by bFGF suggesting initiation of an autocrine signaling loop that promotes STAT3 activation, bFGF expression, and angiogenesis [128]. Hepatocyte growth factor (HGF) was discovered as a neuronal survival factor but also induces proliferation, migration, and tumor angiogenesis. Introduction of an active Src was seen to induce HGF expression with concomitant STAT3 activation in murine mammary adenocarcinoma cells [63]. The gene for HGF was found to contain STAT3 binding sites and direct association of these sites by STAT3 was observed by EMSA [63]. A later study confirmed binding of STAT3 to the HGF gene promoter via ChIP in response to IL-6 in a rat pancreatic β-cell line [64]. Induction of tumor angiogenesis supports tumor metastasis by delivering nutrients to allow for cell survival but also the new vasculature provides an opportunity for migratory cells to enter the circulation and travel to other tissues for implantation. STAT3 induction of angiogenesis and proteases that support angiogenesis and cell invasion together make a lethal combination in tumor biology.

### 2.6. Cell Signaling

While JAK-STAT3 signaling could be described as proliferation or survival promoting signaling due to some of the already discussed STAT3 target genes, STAT3 can also directly up regulate genes to promote activation of other signaling pathways to induce proliferation and survival. AKT is a serine/threonine kinase commonly activated by the PI3K signaling pathway and participates in many functions of the cell including proliferation, cell growth, and glucose metabolism among many others [129]. AKT protein levels were seen to be increased with treatment of IL-6 and introduction of an active STAT3 induced transcriptional activation of the *AKT1* gene in 293 cells [65]. Several predicted STAT3 binding sites were found in the proximal portion of the *AKT1* promoter and multiple ChIP assays confirmed STAT3 was bound to the *AKT1* promoter [65]. STAT3 has also been shown to directly regulate the serine/threonine kinase PIM-1, which is a proto-oncogene that regulates several cellular functions including proliferation [130]. One study found that expression of a dominant negative STAT3 resulted in Pim-1 downregulation in murine hematopoietic cells and human CD4^+^ T lymphoma cells [131]. Another study confirmed that Pim-1 is a direct STAT3 target as STAT3 was able to the bind the GAS sequence in the Pim-1 gene promoter in T lymphocytes [7]. A more recent study confirmed these results with ChIP assays in rat microglia cells [66].

Both AKT and PIM-1 are intracellular kinases but STAT3 can also regulate membrane receptors that initiate cell signaling pathways. Tumor necrosis factor alpha (TNF-α) acts on TNF-R1 and TNF-R2 to exert actions on cells. TNF-R1 mediates the pro-apoptotic effects of TNF-α by activation of the intracellular death domain inherent in TNF-R1 whereas TNF-R2 lacks this death domain and promotes cell proliferation in cancer cells [132]. IL-6 was seen to induce expression of TNF-R2 in human colorectal adenocarcinoma cells and further analysis revealed two STAT3 binding sites in the gene promoter [67]. Further assays showed STAT3 could directly associate with the TNF-R2 gene promoter to regulate its activation [67]. Sphingosine-1-phosphate (S1P) interacts with the S1P receptor 1 (S1P-R1), a G-protein coupled receptor, to regulate many cellular functions relevant to cancer including proliferation, migration, invasion, and angiogenesis among others [133]. The S1P-R1 protein was observed to be increased in murine B cell tumors with active STAT3 whereas S1P-R1 was down regulated in tumors lacking STAT3 [68]. STAT3 expression was shown to drive transcriptional activation of the S1P-R1 gene and mutation of predicted STAT3 binding sites in the promoter ablated this activation [68]. STAT3 was ultimately shown to directly bind and regulate the gene promoter for S1P-R1 [68]. Interestingly, this study also observed that activation of S1P-R1 was shown to activate STAT3 forming an autoregulatory loop to further induce expression of the receptor and downstream signaling.

Lastly, STAT3 has been shown to regulate the gene for mucin-1 (MUC-1). MUC-1 is a member of the mucin family of glycoproteins and is a transmembrane protein with the extracellular portion of the protein affording a layer of protection of epithelial linings [134]. MUC-1 is frequently overexpressed in breast cancer and interacts with receptor tyrosine kinases, such as EGFR, to promote their activation and upregulation of proliferative signaling [134]. Upon search of the *MUC1* gene to identify regulatory proteins, a STAT3 binding site was identified [70]. IL-6 induced transcriptional activation of the MUC-1 gene in human mammary ductal carcinoma cells but this induction did not occur when the STAT3 site was mutated [70]. This study also found that STAT3 could directly associate with a STAT3 binding site within the *MUC1* promoter in response to IL-6 but that STAT1 bound to the MUC-1 promoter in response to IFN-γ [70]. These data suggest an even further variety of ways in which STAT3 can support tumor growth. STAT3 appears to support tumor growth on multiple fronts of cell biology and in multiple stages of tumor development and progression. However, despite the overwhelming amount of information that supports STAT3 as an oncogene, there is some evidence that STAT3 can also suppress tumor growth.

## 3. Tumor Suppressing Functions of STAT3

As described above, STAT3 can directly regulate a host of genes that ultimately support tumor growth and progression such as genes involved in cell signaling, tumor angiogenesis, and metastasis. However, as this article series is making clear, STAT3 also has several tumor suppressive functions as a transcription factor including regulating genes for other tumor suppressing transcription factors and genes that suppress cell proliferation and survival as well as tumor metastasis.

### 3.1. Transcription Factors

The FOX family of transcription factors is characterized as containing the forkhead box domain allowing DNA binding and this large family has ability for gene induction and repression. The FOXO class of FOX transcription factors are a family of tumor suppressors as they block entry to the cell cycle and are inactivated by phosphorylation by several kinases including AKT and CDK1 [135]. STAT3 has been shown to up regulate both FOXO1 and FOXO3A in CD4^+^ T cells [71]. The genes for FOXO1 and FOXO3A were found to have STAT3 binding sites and ChIP assays confirmed direct association of STAT3 with both gene promoters [71]. Within another FOX class, the FOXP3 transcription factor was originally discovered as the master regulator in regulatory T cells [136]. However, further evidence indicates FOXP3 is also a tumor suppressor that is down regulated in several types of carcinomas and has the ability to repress several oncogenes including HER2 and c-MYC and up regulate the tumor suppressor p21^WAF1/CIP1^ [136]. FOXP3 expression was found to be increased by IL-2-induced activation of STAT3 in 293 cells [72]. While they did not confirm STAT3 directly bound the *FOXP3* gene, mutation of predicted STAT3 binding sites within the first intron of the *FOXP3* gene significantly reduced STAT3-induced transcriptional activation of FOXP3 [72]. 

Necdin, a member of the melanoma antigen gene (MAGE) family, is a transcriptional repressor that facilitates entry into the cell cycle [137]. Aside from its transcriptional role, Necdin also has the ability to physically associate with p53 to suppress p53-dependent apoptosis promoting survival of post-mitotic neurons [138]. Necdin was identified as a potential STAT3 target via microarray experiments and further investigation revealed that STAT3 could directly bind the gene promoter for Necdin leading to downregulation of Necdin levels in v-Src-expressing NIH-3T3 cells [73]. Together, these studies point to the potentially opposing role STAT3 can have on tumor growth by regulating these tumor suppressing transcription factors.

### 3.2. Survival and Metastasis

Uncontrolled proliferation and cell survival are key factors in tumorigenesis of primary tumors and metastasis of cells from this primary tumor worsens patient prognosis and treatment options. STAT3 can regulate several tumor suppressing genes in these areas.

The cyclin-dependent kinases (CDKs) have endogenous inhibitors and CDK4/6 is specifically inhibited by the protein p21^WAF1/CIP1^. STAT3 has been shown to directly bind the gene promoter for p21^WAF1/CIP1^ inducing its expression in multiple cell types including epidermoid carcinoma, colorectal carcinoma, and bone osteosarcoma cells [74,75,76]. One of these studies identified NcoA/SRC1a as co-factors that bind to the p21^WAF1/CIP1^ gene promoter with STAT3 [76]. 

The regulation of p21^WAF1/CIP1^ is a more direct effect of STAT3 on regulating the cell cycle and proliferation. STAT3 can also regulate expression of subunits of the PI3K complex to suppress cell survival. The class IA PI3K complex is made up of a catalytic subunit (p110α, p110β, or p110δ) and a regulatory subunit (p85α, p85β, p55α, p55γ, or p50α) that combines to respond to receptor tyrosine kinases. These PI3Ks phosphorylate phosphoinositols allowing recruitment of other kinases, such as AKT, leading to their activation and downstream signaling that typically results in cell survival among other tumor supporting functions. During mammary gland involution, STAT3 has previously been shown to promote apoptosis leading to post-lactation gland regression [139]. One study found that STAT3 was activated whereas AKT was inactivated in mouse mammary glands undergoing involution [77]. Targeted deletion of STAT3 in these mammary glands resulted in decreased expression of the p55α and p50α PI3K subunits [77]. Similarly, STAT3 activation induced expression of p55α and p50α and this correlated with increased apoptosis during involution [77]. STAT3 binding sites were found in the gene promoters for both p55α and p50α and a ChIP assay confirmed association of STAT3 with these sites [77]. Ultimately, STAT3 was found to promote apoptosis in these cells due to upregulation of p55α and p50α, which inhibited PI3K signaling and AKT activation reducing cell signaling for survival [77]. However, this relationship did not extend to embryonic stem cells suggesting this mechanism may be specific to the mammary tissue or during the involution stage and gland regression [77].

STAT3 can, therefore, have effects that both promote and suppress tumor growth. STAT3 seemingly has many more functions that support tumor growth than to suppress tumor growth. However, there are also a great number of genes that STAT3 regulates that have dual roles in tumor growth.

## 4. Dual Functions by STAT3 in Tumor Growth

To this point, STAT3 has been discussed purely as supporting tumor growth or purely as suppressing tumor growth. However, STAT3 has also been shown to (1) upregulate and downregulate specific genes involved in tumor growth or (2) regulate genes that may themselves support or suppress tumor growth depending on the context.

### 4.1. Tumor Immune Function

STAT3 can directly regulate the genes for multiple cytokines, which can lead to enhanced or suppressed tumor immune function. In studies identifying STAT3 as a tumor immune suppressor, one of the observations was decreased levels of IL-6 with increased STAT3 expression in NIH-3T3 cells and murine macrophages [78,80]. However, it was later observed that STAT3 can bind to the gene promoter and up regulate IL-6 in response to IL-32α in a manner dependent on PKCε [79]. These different results may be a result of cellular context as PKCε was a co-factor required for STAT3 binding and upregulation of the IL-6 gene. Similarly, expression of a STAT3 dominant negative has been shown to increase TNF-α levels in murine macrophages [80] whereas another study observed that mutation of the STAT3 binding sites in the gene promoter for TNF-α reduced activation of the TNF-α gene [81]. Although an AP-1 binding site was also found in the gene promoter for TNF-α, AP-1 did not appear to play a role in STAT3-mediated gene activation from the data presented [81].

Similar to these genes, STAT3 also has dual regulation of IFN-γ and RANTES. IFN-γ is a cytokine that promotes immune function by activating NK cells and promoting Th1 cell differentiation among other functions. Studies that found STAT3 suppressed tumor immune surveillance found that STAT3 presence resulted in decreased levels of IFN-γ in murine macrophages [78,80]. However, another study found that IL-12 could activate mTOR and STAT3 phosphorylation and ultimately promote IFN-γ production dependent on mTOR activity [82]. This study also identified STAT3 binding sites within the gene for IFN-γ and confirmed direct binding of STAT3, along with STAT4 and c-Jun, to the gene promoter for IFN-γ in response to IL-12 in human T cells [82]. RANTES is another chemokine that supports immune function by attraction of T cells and monocytes and inducing T cell proliferation. Similar to IFN-γ, RANTES was observed to be negatively regulated by STAT3 expression in murine macrophages [78,80]. However a later study showed that an unphosphorylated form of STAT3, lacking ability for phosphorylation on Y705, could induce RANTES expression by directly interacting with the gene promoter for RANTES in a complex with the p65 subunit of NF-κB [83]. Together these data show that STAT3 can both up regulate and down regulate the genes for cytokines that support immune function to suppress tumor growth. The differential regulation of these genes by STAT3 appears to be related to what STAT3 co-factors are present and even what form of STAT3 is predominantly present (*i.e.*, phosphorylated *vs.* unphosphorylated).

STAT3 also regulates several other genes that have immune functions that are not as consistent. C-reactive protein (CRP) is an acute-phase protein that has a rapidly increased expression following acute inflammation and CRP activates the complement system. Circulating CRP levels are associated with an increased cancer risk although it has been concluded this link is not causal [140]. However there are more questions regarding the role the complement system plays in cancer as it has long been thought to promote immune surveillance of developing tumors but it has also been shown to promote tumor growth [141]. Treatment of human hepatocellular carcinoma cells was shown to induce CRP expression, which was dependent on STAT3 binding sites within the *CRP* promoter [84]. Furthermore, direct association of STAT3 with the *CRP* promoter was also shown under these conditions [84].

STAT1 is another protein shown to be regulated by STAT3 but may support or suppress tumor growth. STAT1, another member of the STAT family, is activated by IFN-γ and has multiple tumor suppressor functions such as enhancement of immune surveillance and promotion of apoptosis [142]. However, STAT1 has also been shown to support inflammation-associated gastric tumorigenesis [143]. Our lab observed increased STAT1 expression in human mammary adenocarcinoma in the presence of nuclear EGFR, which synergized with co-expression of STAT3 to further enhance STAT1 levels [85]. The *STAT1* promoter was found to have several STAT3 binding sites and mutation of these sites suppressed STAT1 gene activation by STAT3 and EGFR co-expression [85]. We further showed that regulation by STAT3 and EGFR was the result of a direct association of STAT3 and EGFR with the *STAT1* promoter in a STAT3-dependent manner [85]. Whether STAT3-mediated upregulation of STAT1 and CRP actually supports or suppresses tumor growth is not clear to this point despite both STAT1 and CRP potentially having both pro- and anti-tumor activity.

Lastly, STAT3 has a significant impact on tumor immune function through its role in the development of Th17 T helper cells. Th17 cells were identified by their large production of IL-17, which is unique from Th1 and Th2 cells [144,145]. The function of Th17 cells in tumor immunity is not completely understood as some results suggest pro-tumor activity while others suggest anti-tumor activity [146,147]. Six transcription factors have been identified as critical for Th17 differentiation: STAT3, RORγt, RORα, IRF4, BATF, and HIF-1α [147]. Deletion of STAT3 in mouse CD4^+^ naïve T cells prevents their differentiation into Th17 cells whereas expression of an active STAT3 promotes Th17 differentiation [83,86]. Using ChIP coupled with massive parallel sequencing (ChIP-Seq), STAT3 was identified to bind to the gene promoters for RORγt, RORα, BATF, and IRF4 [86]. RORγt is considered the master regulator of Th17 cell differentiation and STAT3 was found to bind within the first intron of the mouse gene leading to increased expression and promotion of Th17 differentiation [86]. It was mentioned above that STAT3 could also directly up regulate HIF-1α gene expression and protein half-life [28] possibly indicating, although STAT3 regulation of HIF-1α was observed in melanoma cells, that STAT3 can directly regulate all six transcription factors involved in Th17 cell differentiation. This study also identified STAT3 binding to the genes for IL-23R and IL-6Rα, which supports Th17 development as both IL-6 and IL-23 promote Th17 differentiation [83,86]. In addition, STAT3 was also found to bind the genes for IL-17A and IL-17F suggesting STAT3 also participates in the production of IL-17 that led to the identification of Th17 cells [86]. Although STAT3 promotes Th17 development, Th17 cells themselves may support or suppress tumors. Therefore the regulation of Th17 development by STAT3 is an example of a family of genes regulated by STAT3 that drive cell development and affect tumor immune function.

### 4.2. Other

STAT3 has also been shown to have differing effects on tumor growth through regulation of TIMPs. The MMP family has natural inhibitors termed the tissue inhibitor of metalloproteinases (TIMPs) that suppress the protease function of MMPs. STAT3 has been shown to directly target the TIMP-1 gene as IL-6 has been observed to up regulate TIMP-1 levels in a STAT3-dependent manner in human hepatocellular carcinoma cells as well as human fibroblasts [89]. A STAT3 binding site was found in the gene for TIMP-1 and deletion of the STAT3 binding sites completely ablates TIMP-1 activation by IL-6 [89]. This site was also confirmed to bind STAT3 using EMSA but further analysis of gene activation showed that binding of AP-1 was required for full activation by STAT3 [89]. A later study confirmed the TIMP-1 gene as a STAT3 target using ChIP-Seq in CD4^+^ T cells [88]. Considering MMPs promote cancer cell invasion, metastasis, and angiogenesis, it would seem that STAT3 upregulation of an endogenous inhibitor to MMPs is another example of STAT3 showing both oncogenic and tumor suppressor functions. However, TIMP-1 has been shown to have functions outside of MMP inhibition related to growth stimulation, apoptosis suppression, and induction of angiogenesis [148]. Furthermore, TIMP-1 is frequently overexpressed in breast cancer and this is associated with a poor prognosis [148]. STAT3 upregulation of TIMP-1 provides a clear illustration of a gene up regulated by STAT3 but may support or suppress tumor growth depending on the context in which it is expressed.

Another example of a gene that is primarily up regulated by STAT3 but may support or suppress tumor growth is JunB. As mentioned above, JunB is in the family of Jun proteins that make up the AP-1 transcription factor. STAT3 was found to bind to predicted STAT binding sequences within the *JUNB* promoter in response to IL-6 treatment in HepG2 hepatocellular carcinoma cells [26,90]. While there is a great deal of evidence suggesting AP-1 supports tumor growth, there are also indications that JunB can act as a tumor suppressor in certain contexts [92]. The role of STAT3 in tumor suppression by regulation of JunB has not been made clear but this is an example of a STAT3-regulated gene that may support or suppress tumor growth.

Nitric oxide (NO) is produced in cells by nitric oxide synthases (NOS), in which there are three isoforms: endothelial (eNOS), neuronal (nNOS), and inducible (iNOS) [149]. The expression of eNOS and nNOS are relatively limited to a small number of tissues and are calcium-dependent whereas iNOS is calcium-independent and can be expressed in a large variety of tissues [149]. The expression of iNOS is associated with several types of cancer and the produced NO has many pro-tumorigenic functions such as activation of signaling pathways and inhibition of DNA repair [149]. However, there are instances in which NO suppresses tumor growth such as enhanced activity of Fas and inhibition of NF-κB [149]. The iNOS gene was identified as a potential target gene for the nuclear EGFR-STAT3 complex [18]. Further analysis of the gene for iNOS found multiple STAT3 binding sites and mutation of these sites reduced transcriptional activation by the STAT3-EGFR complex [18]. Both EMSA and ChIP assays confirmed that STAT3 and EGFR bind to and regulate the gene promoter for iNOS in epidermoid carcinoma cells [18]. This study suggested STAT3 regulation of iNOS supported growth of malignant tumor cells. However, iNOS and NO also have potential tumor suppressing functions and the role of STAT3 in these have not been clearly addressed.

STAT3 can also regulate the dual specificity phosphatase CDC25A, which can remove phosphate from serine, threonine, or tyrosine residues. CDC25A is required for cell cycle progression from the G1 to the S phase [150]. IL-6 induced expression of CDC25A in a STAT3-dependent manner in human hepatocellular carcinoma cells [91]. A ChIP assay found that STAT3 was directly bound to *CDC25A* promoter in a manner dependent on c-Myc binding [91]. In addition to this mechanism of STAT3-mediated upregulation of CDC25A, this study also observed a mechanism whereby STAT3 suppresses CDC25A. Hydrogen peroxide was seen to induce interaction between STAT3 and retinoblastoma (Rb) protein and this complex occupied the *CDC25A* promoter in the same proximal region as the STAT3-c-Myc complex to repress expression [91]. This is further evidence suggesting the cellular context may play a significant role in whether STAT3 up regulates or down regulates genes in which STAT3 has dual regulation and, consequently, whether STAT3 supports or suppresses tumor growth.

## 5. Conclusions

Extensive efforts have been and are being actively invested in developing and testing STAT3-targeted therapy against a variety of human cancers. To date, the clinical testing of the STAT3-targeted therapy is still in the early stage with promising results being reported. To maximize these clinical efforts, a deeper understanding of the STAT3 pathway is urgently needed. To meet this need, additional investigations are required to decipher the full spectrum of STAT3 target genes in the context of different cellular environment and various types of human cancers, gain mechanistic insights into how STAT3 is regulated, deepen our understanding of the existing and novel signaling pathways that crosstalk with STAT3, and to identify novel cellular processes that can be regulated by STAT3 but may have been overlooked.
